# Decomposition of high-frequency electrical conductivity into extracellular and intracellular compartments based on two-compartment model using low-to-high multi-*b* diffusion MRI

**DOI:** 10.1186/s12938-021-00869-5

**Published:** 2021-03-25

**Authors:** Mun Bae Lee, Hyung Joong Kim, Oh In Kwon

**Affiliations:** 1 Department of Mathematics, Konkuk University, 05029 Seoul, South Korea; 2 Department of Biomedical Engineering, Kyung Hee University, 02447 Seoul, South Korea

**Keywords:** Magnetic resonance electrical property tomography, High-frequency conductivity decomposition, Multi-*b* diffusion weighted imaging, Low-frequency conductivity tensor, Random forest

## Abstract

**Background:**

As an object’s electrical passive property, the electrical conductivity is proportional to the mobility and concentration of charged carriers that reflect the brain micro-structures. The measured multi-*b* diffusion-weighted imaging (M*b*-DWI) data by controlling the degree of applied diffusion weights can quantify the apparent mobility of water molecules within biological tissues. Without any external electrical stimulation, magnetic resonance electrical properties tomography (MREPT) techniques have successfully recovered the conductivity distribution at a Larmor-frequency.

**Methods:**

This work provides a non-invasive method to decompose the high-frequency conductivity into the extracellular medium conductivity based on a two-compartment model using M*b*-DWI. To separate the intra- and extracellular micro-structures from the recovered high-frequency conductivity, we include higher *b*-values DWI and apply the random decision forests to stably determine the micro-structural diffusion parameters.

**Results:**

To demonstrate the proposed method, we conducted phantom and human experiments by comparing the results of reconstructed conductivity of extracellular medium and the conductivity in the intra-neurite and intra-cell body. The phantom and human experiments verify that the proposed method can recover the extracellular electrical properties from the high-frequency conductivity using a routine protocol sequence of MRI scan.

**Conclusion:**

We have proposed a method to decompose the electrical properties in the extracellular, intra-neurite, and soma compartments from the high-frequency conductivity map, reconstructed by solving the electro-magnetic equation with measured B1 phase signals.

## Background

Using a conventional MRI scanner without any external electrical stimulation, magnetic resonance electrical properties tomography (MREPT) techniques have been developed and successfully recover the conductivity distribution at Larmor-frequency (about 128 MHz at 3 T ) [1–4]. The electrical conductivity of biological tissues is proportional to the apparent concentration and mobility of ions in the intracellular and extracellular compartments. As the separated form of electrical conductivity of biological tissues, the low-frequency conductivity (< 1 kHz) is dominantly influenced by the apparent concentration and mobility of ions in the extracellular compartment.

To decompose the high-frequency conductivity into the extracellular and intracellular compartments using a conventional clinical MRI scanner, it requires micro-structural parameters including the extracellular volume fraction (EVF), distributions of electrical charged molecules, and diffusion coefficient coefficients in the extracellular and intracellular compartments [5, 6]. For the model-based micro-structure imaging based on the tissue micro architecture in the brain, the micro-structural parameters for the intracellular and extracellular compartments have been studied, related with specific tissue micro-structure features from M*b*-DWI data [7–9]. Although a two compartment model is the simplest form, the determination of micro-structural parameters from measured decay MR DWI signals with respect to *b*-value is ill-posed because the estimation of parameters from the combination of smooth exponential curves is sensitive to noise in the measured DWI data. To stabilize the ill-posedness, two compartment models typically assume some restrictions: intrinsic diffusivity and/or water diffusing in elongated cellular fibres, based on the ball-and-stick model [10, 11].

Various exponential diffusion models have been proposed to describe signal attenuation with DWIs using more than two *b*-values [12–17]. Using the exponential diffusion models, an electrodeless method providing the low-frequency electrical property imaging without any external hardware was proposed [6, 17–19]. The proposed methods mainly focus on separating the ion mobility and concentration in the extracellular space (ECS) from the recovered high-frequency conductivity. Although, without injection of external currents, it is a promising work to separate the high-frequency conductivity (combined electrical properties from the extra- and intra-celluar compartments) into the low-frequency conductivity (electrical properties from the extracellular compartment), the developed methods still have difficulties in distinguishing the hindered diffusion of free water molecules and the diffusion-limited compartment [17, 19]. For separating the low-frequency conductivity from the high-frequency conductivity, we need to quantify the micro-structures of extracellular compartment. However, typical two compartment models based on the ball-and-stick model consider the diffusion signals from soma or other large cellular domains as those from the extracellular compartment. The reconstructed low-frequency conductivity using the ball-and-stick model overestimates EVF and, as a result, causes the biased conductivity values, especially in the gray matter region.

Recently, a biophysical model has been proposed for apparent cell body (soma) and neurite density imaging (SANDI), which tries to recover the soma size and density in addition to neurite density [9]. In particular, in ECS, to decompose the electrical properties of each compartment, it is important to distinguish the micro-structural characteristics of the soma size and density belonging to the intracellular compartment. The SANDI model needs to include the direction-averaged DWI signal at high *b*-values ($\ge$ 3000 s/$\hbox {mm}^2$) to detect the apparent soma size and density. Combining with the estimated DWI data for the higher *b*-values, we apply the SANDI model to estimate the micro-structures of extracellular compartment and use it to identify the characteristics of low-frequency conductivity.

To determine the micro-structural parameters of biological tissues using the measured M*b*-DWI data, we use the random forest regression, an ensemble machine learning algorithm by constructing a multitude of decision trees at training time [20, 21]. The applied machine learning method builds a forest of uncorrelated trees, combined with randomized node optimization and bootstrap aggregating [22].

To demonstrate the electrical property decomposition from the recovered high-frequency conductivity, we generate the high-frequency conductivity using the convection-reaction partial differential equation with a small regularization parameter [1]. The recovered high-frequency conductivity reflects the combined electrical properties by the intra- and extra-cellular compartments using a routine protocol sequence of MRI scan.

To validate the proposed method, a conductivity phantom including a giant vesicle suspension was conducted as a model for the membranes of biological cells. The giant vesicles were cell-like materials with thin insulating membranes to validate the two compartment model including both extracellular and intracellular spaces. We conducted human experiments by comparing the results of reconstructed conductivity of extracellular medium and the conductivity in the intra-neurite and intra-cell body. We extracted the apparent total ion concentration from the high-frequency conductivity map and the estimated micro-structural parameters. The phantom and human experiments indicate that the total ion concentration and the extracellular diffusion tensor can predict the extracellular electrical properties without externally injected currents. The accuracy and precision of the reconstructed low-frequency conductivity distribution in the extracellular compartment were evaluated.

## Results

### Giant vesicle phantom experiment

Giant membrane vesicles dispersed in aqueous solution were prepared as described in [23]. Giant vesicles are a model membrane system for the investigation of lipid membranes. In a 1L round-bottom flask containing 3 mL of chloroform and 400 μm of methanol, 2 mL of phospholipids dissolved with a chloroform solution of 30 mg/mL concentration under argon atmosphere. A 20 mL volume of distilled water or 0.75% NaCl solution was carefully added not to disturb the interface between the aqueous phase and organic solution phase. The flask was installed to a rotary evaporator to remove organic solvent at 47 °C under vacuum for 20 min at 10 rpm and then followed by another 20 min at 60 rpm. During evaporation of organic solvents, phospholipids were assembled to form giant vesicles. The resultant aqueous solution containing giant vesicles were centrifuged at 1500 rpm for 10 min. The volume fraction of the giant vesicles was about 80 to 90% by visual observations. The mean and standard deviation of the diameters of the giant vesicles were 13 ± 4.7 μm.

**Fig. 1:**
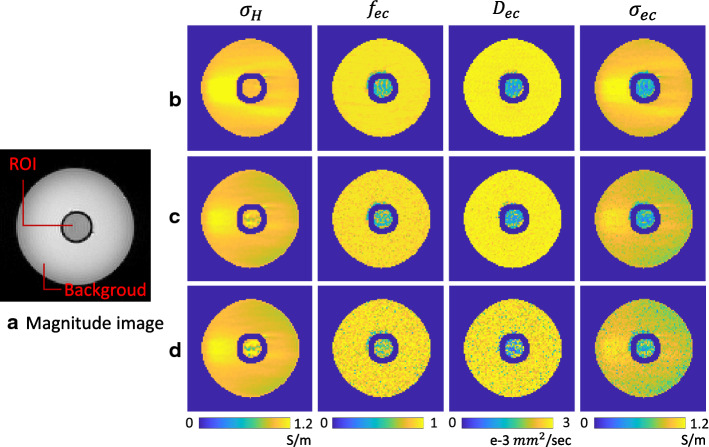
**a** Magnitude image. **b**–**d** Estimated high frequency conductivity,$\sigma _H$, extracellular volume fraction, $f_{ec}$, extracellular diffusivity, $D_{ec}$, and extracellular conductivity, $\sigma _{ec}$ for different noise levels of ($\hbox {SNR}_P$, $\hbox {SNR}_D$). **b** ($\infty ,\infty$), **c** (100,50), **d** (50,10))

The conductivity phantom was constructed, which comprised two compartments of electrolyte (Background) and giant vesicle suspension (ROI) (Fig. 1a). The background was an NaCl solution of 3 g/L and ROI was filled with the giant vesicles suspension. The giant vesicle suspension was a mixture of the same NaCl solution of background and giant vesicles. In the giant vesicle suspension, the ratio of NaCl solution and giant vesicle was the same, which means that the volume fraction of the intracellular space was about 0.4–0.45.

A 9.4 T research MRI scanner (Agilent Technologies, USA) was used with a single-channel mouse body coil. The multi-spin-echo pulse sequence was used to acquire B1 phase maps to reconstruct high frequency conductivity images of the phantom. The imaging parameters were as follows: TR/TE = 2200/22 ms, number of echoes (NE) = 6, number of excitation (NEX) = 5, slice thickness = 0.5 mm, field-of-view (FOV) = 65$\times$65 $\hbox {mm}^2$, flip angle = 90${^\circ }$, image matrix size = 128$\times$128$\times$6, and scan time = 23 min. Diffusion weighted MR imaging was separately performed using the single-shot spin-echo echo planar imaging sequence. The imaging parameters were as follows: TR/TE = 2000/70 ms, $\delta /\Delta$ = 6/53.8 ms, NEX = 2, slice thickness = 0.5 mm, FOV = 65$\times$65 $\hbox {mm}^2$, flip angle = 90$^{\circ }$, and image matrix size = 128 × 128 × 6. The number of directions of the diffusion-weighting gradients was 30 with *b*-values of 1000, 2200, 3000 and 3600 s/$\hbox {mm}^2$. The scan time was about 32 min. An additional conventional $\hbox {T}_1$ weighted scan of 5 min was included for anatomical reference.

The conductivity values of the giant vesicle suspension in ROI (Fig. 1a) were measured by using an impedance analyzer (SI1260A, AMETEK Inc., UK). The conductivity values were 0.57 S/m at 1 kHz and 1.05 S/m at 5 MHz.

Figure 1b shows the estimated high-frequency conductivity, $\sigma _H$, extracellular volume fraction, $f_{ec}$, extracellular diffusivity, $D_{ec}$, and extracellular conductivity, $\sigma _{ec}$, respectively. Table 1 shows the mean and standard deviation values of the estimated high frequency conductivity, extracellular volume fraction, extracellular diffusivity, extracellular ion concentration and extracellular conductivity within ROI.

**Table 1:** Mean and standard deviation values of the high frequency conductivity, $\sigma _H$, extracellular volume fraction, $f_{ec}$, extracellular diffusivity, $D_{ec}$, extracellular ion concentration, ${\bar{c}}_{ec}$, and extracellular conductivity, $\sigma _{ec}$ within ROI

	$\sigma _H$	$f_{ec}$	$D_{ec}$	${\bar{c}}_{ec}$	$\sigma _{ec}$	Measured conductivity	
ROI	1.01 ± 0.02	0.59 ± 0.14	1.36 ± 0.43	0.71 ± 0.15	0.54 ± 0.15	0.57 (1 kHz)	1.05 (5 MHz)

Conductivity values measured using the impedance analyzer are shown in the last two columns

The estimated high-frequency conductivity was $\sigma _H=1.01\pm 0.02$ S/m in ROI, which is close to the conductivity value 1.05 S/m measured using the impedance analyzer at 5 MHz in ROI. We used the diffusion term $c=0.05$ to stabilize the convection-reaction partial differential equation in (3). Since the volume fraction of the giant vesicles after the centrifugation was about 80% to 90% and the ratio of NaCl solution and giant vesicles was same in ROI, the controlled extracellular volume fraction was about 0.55–0.60 in ROI. The value of $f_{ec}$ in ROI was 0.59±0.14 as expected.

To estimated the extracellular ion concentration, ${\bar{c}}_{ec}$, in (12), we assume that the intra-neurite ion concentration and soma ion concentration are same, and further assume that ${\bar{c}}_{in}=\beta {\bar{c}}_{ec}$ for some constant $\beta$. Since we used the same electrolyte for both inside and outside of the giant vesicle, the ratio of ion concentrations in the intracellular and extracellular compartments, $\beta$, was 1. The extracellular ion concentration ${{\bar{c}}_{ec}}$ can be estimated as

1 $$\begin{aligned} {\bar{c}}_{ec}=\frac{\sigma _H}{ \beta (f_{ne} D_{in} + f_{so} D_{is}) + f_{ec} D_{ec} } \end{aligned}$$

With the estimated high-frequency conductivity and the parameters of the SANDI model for brain micro-structure, we recovered the extracellular ion concentration, ${\bar{c}}_{ec}$, extracellular conductivity, $\sigma _{ec}$. $\sigma _{ec}$ = 0.54 ± 0.15 S/m in ROI is close to the measured conductivity value 0.57 S/m at 1 kHz.

To verify the proposed method, we conducted two cases. We artificially destroyed the measured signals by adding random noise and motion artifacts. For noisy data, we added the zero-mean Gaussian random noise to both the phase data and diffusion weighted signal. The noise standard deviation were calculated by $\langle S \rangle$/($\sqrt{2}$ SNR), where $\langle S \rangle$ is the average signal (phase signal and the signal obtained without diffusion gradient) amplitude and SNR is the signal-to-noise ratio in MR magnitude images. For the phase data, $\hbox {SNR}_P$ values 200, 100, and 50 were employed. For the diffusion weighted signal, we assumed that $\hbox {SNR}_D$ of the signal obtained without diffusion gradient, $S_0$, were 100, 50, and 10. The random noise added to $S_0$ was added to each $S_b$.

Figure 1c, d shows images of the estimated high frequency conductivity, $\sigma _H$, extracellular volume fraction, $f_{ec}$, extracellular diffusivity, $D_{ec}$, and extracellular conductivity, $\sigma _{ec}$ for ($\hbox {SNR}_P$, $\hbox {SNR}_D$) = (100,50) and (50,10). Table 2 shows the mean and standard deviation values of the estimated high frequency conductivity, extracellular volume fraction, extracellular diffusivity, extracellular ion concentration and extracellular conductivity within ROI for the chosen noise levels. The proposed method stably reconstructed the conductivity values when $\hbox {SNR}_P$ and $\hbox {SNR}_D$ were above 100.

**Table 2:** Mean and standard deviation values of the high frequency conductivity, $\sigma _H$, extracellular volume fraction, $f_{ec}$, extracellular diffusivity, $D_{ec}$, extracellular ion concentration, ${\bar{c}}_{ec}$, and extracellular conductivity, $\sigma _{ec}$ within ROI for different noise levels of ($\hbox {SNR}_P$, $\hbox {SNR}_D$)

	(200,100)	(200,50)	(200,10)	(100,100)	(100,50)	(100,10)	(50,100)	(50,50)	(50,10)
$\sigma _H$	1.00 ± 0.06	1.00 ± 0.06	1.00 ± 0.06	1.02 ± 0.12	1.02 ± 0.12	1.02 ± 0.12	1.09 ± 0.29	1.09 ± 0.29	1.09 ± 0.29
$f_{ec}$	0.61 ± 0.15	0.60 ± 0.16	0.70 ± 0.16	0.61 ± 0.15	0.60 ± 0.16	0.70 ± 0.16	0.61 ± 0.15	0.60 ± 0.16	0.70 ± 0.16
$D_{ec}$	1.42 ± 0.51	1.50 ± 0.55	1.68 ± 0.83	1.42 ± 0.51	1.50 ± 0.55	1.68 ± 0.83	1.42 ± 0.51	1.50 ± 0.55	1.68 ± 0.83
${\bar{c}}_{ec}$	0.70 ± 0.17	0.68 ± 0.18	0.72 ± 0.34	0.71 ± 0.19	0.69 ± 0.20	0.73 ± 0.34	0.76 ± 0.29	0.74 ± 0.28	0.78 ± 0.38
$\sigma _{ec}$	0.56 ± 0.16	0.55 ± 0.16	0.67 ± 0.16	0.57 ± 0.17	0.56 ± 0.17	0.68 ± 0.17	0.61 ± 0.23	0.60 ± 0.24	0.72 ± 0.23

**Fig. 2:**
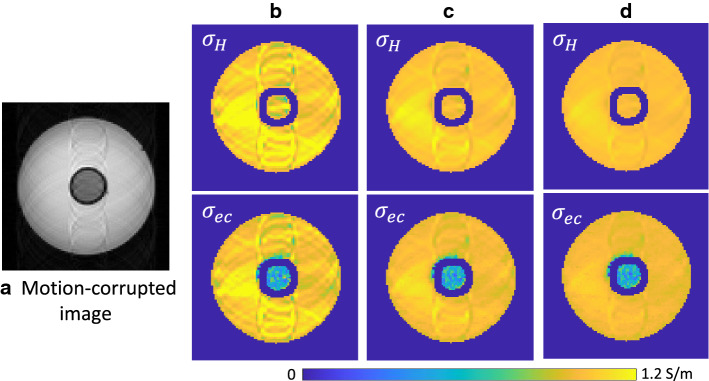
**a** Magnitude image with motion artifacts. **b**–**d** Estimated high frequency conductivity, $\sigma _H$, and intracellular conductivity, $\sigma _{ec}$ for different diffusion term *c* in (3). ((b): $c=0.1$, **c** $c=0.5$, **d** $c=1$)

To investigate the impact of subject motion, we simulated a sinusoidal motion in the phase-encoding direction with amplitude 1 and a frequency of 2 Hz and obtained a motion-corrupted complex MR signals which were the convolution of the measured complex MR signals with the corresponding point spread function [24]. Figure 2a shows the magnitude image with motion artifacts. Figure 2b–d shows images of the estimated high frequency conductivity, $\sigma _H$, and extracellular conductivity, $\sigma _{ec}$ for the different diffusion term *c* in (3). Since the diffusion term *c* acts as a low-pass filter in the reconstruction algorithm, it was possible to reconstruct the motion-corrected high-conductivity map using a high *c* value. For $c=1$, the values of the estimated high frequency conductivity and extracellular conductivity within ROI were 0.99$\pm 0.02$ and 0.53 ± 0.14, respectively.

### Human experiment

MRI measurements were performed with three healthy volunteers without a documented history of any disease were recruited. The participants were located inside the bore of a 3T MRI scanner with the head coil in transmit and a 32-channel RF head coil (Achieva TX, Philips Medical Systems, the Netherlands). All experimental protocols were approved by the institutional review board of Kyung Hee University (KHSIRB-16-033). All methods were carried out in accordance with the relevant guidelines and regulations and all participants provided written informed consent.

For MREPT imaging experiments, the multi-spin-echo pulse sequence with multiple refocusing pulses was adopted to minimize the measured noise. Before the data acquisition, we applied a volume shimming method with the volume defined to cover the brain region. Imaging parameters were as follows: TR/TE = 1500/15 ms, NE = 6, NEX = 1, slice thickness = 4 mm, FOV = 260$\times$260 $\hbox {mm}^2$, imaging matrix size = 128$\times$128$\times$5, and scan time = 16 min. After MREPT scans, we performed DWI scans using the single-shot spin-echo echo planner imaging (SS-SE-EPI) pulse sequence. We applied the diffusion weighting gradients in 15 directions with 4 *b*-values of 1000, 2200, 3000 and 3600 s/$\hbox {mm}^{2}$, respectively. Imaging parameters were as follows: TR/TE = 2000/70 ms, $\delta /\Delta$ = 21/33 ms, NEX = 2, slice thickness = 4 mm, and acquisition matrix size = 64$\times$64$\times$5. The scan time was about 6.2 min. The matrix size of 64$\times$64$\times$5 was extended to 128$\times$128$\times$5 to match the spatial resolution (2.03$\times$2.03$\times$4  $\hbox {mm}^3$) of MREPT experiment. To increase the spatial resolution with the matrix size of $64\times 64$ to the spatial resolution of $128\times 128$ by reducing scanning times without much loss in resolution or SNR, we used the zero-filling interpolation (ZIP) by zero filling the high spatial-frequency components of the raw *k*-space data, which places the acquired data in the central regions on *k* -space and fills the data of zero in the outer regions. An additional conventional $\hbox {T}_1$ weighted scan of 2 min was included for anatomical reference.

**Fig. 3:**
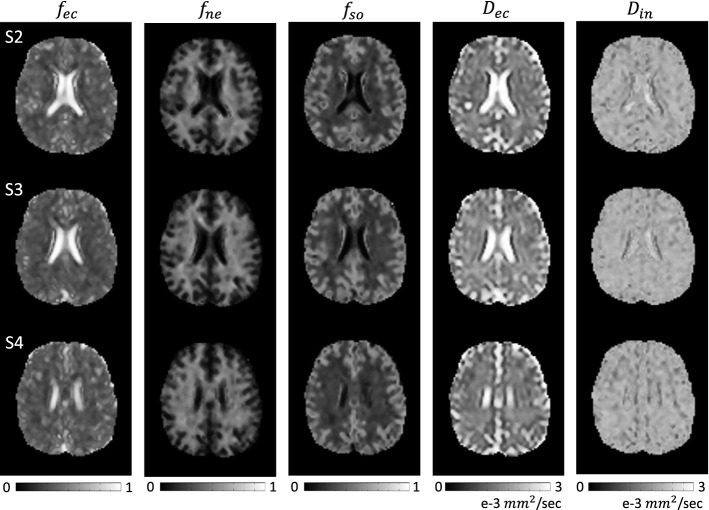
Estimated extracellular volume fraction, $f_{ec}$, intra-neurite volume fraction, $f_{ne}$, soma volume fraction, $f_{so}$, extracellular diffusivity, $D_{ec}$, and intra-neurite diffusivity, $D_{in}$, from the first subject. Imaging slices are shown in the first column

Figure 3 shows the estimated micro-structural parameter maps of SANDI model (5) for the first human subject: the estimated extracellular volume fraction $f_{ec}$, intra-neurite volume fraction $f_{ne}$, soma volume fraction $f_{so}$, extracellular diffusivity $D_{ec}$, and intra-neurite diffusivity $D_{in}$ from the first subject.

**Fig. 4:**
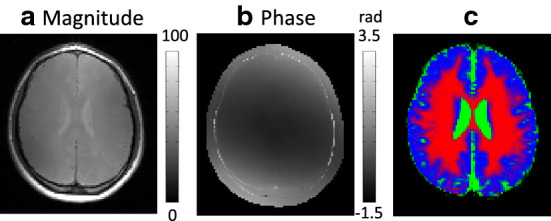
Magnitude, phase, and segmented (CSF, GM, WM) images from the third imaging slice of the first subject

Figure 4a, b shows the MR magnitude and the B1 phase images at the third imaging slice of the first subject. For quantitative analyses, $\hbox {T}_1$ image was segmented into cerebrospinal fluid (CSF), gray matter (GM), and white matter (WM) using the segmentation tool of Statistical Parametric Mapping (SPM 12) [25]. These regions are shown in Fig. 4c.

**Fig. 5:**
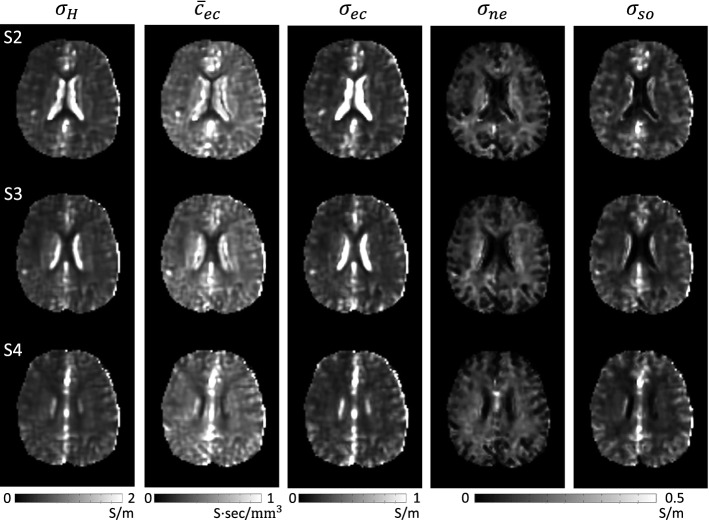
Estimated high-frequency conductivity, $\sigma _H$, extracellular ion concentration, ${\bar{c}}_{ec}$, extracellular conductivity, $\sigma _{ec}$, intra-neurite conductivity, $\sigma _{ne}$, and soma conductivity, $\sigma _{so}$, from the first subject

Figure 5 shows the details of reconstructed conductivity images from the first subject. To estimate the high frequency conductivity, $\sigma _H$, with the acquired transceiver phases of B1 maps, we solved the convection-reaction partial differential equation (PDE) in (3) with the diffusion term $c=0.02$.

To estimate the extracellular ion concentration, ${\bar{c}}_{ec}$, in (12), we set that the ratio $\beta$ for ion concentrations in the intracellular and extracellular compartments to be 0.41 as suggested in [6] by adopting reference values of intracellular and extracellular ion concentrations of four predominant ions ($\hbox {Na}^+$, $\hbox {Cl}^-$, $\hbox {K}^+$, and $\hbox {Ca}^{2+}$). Using the reference ratio value $\beta =0.41$, the extracellular ion concentration ${{\bar{c}}_{ec}}$ can be estimated by (1).

With the estimated high-frequency conductivity and the parameters of the SANDI model for brain micro-structure, we recovered the extracellular ion concentration, ${\bar{c}}_{ec}$, extracellular conductivity, $\sigma _{ec}$, intra-neurite conductivity, $\sigma _{ne}$, and soma conductivity, $\sigma _{so}$.

**Fig. 6:**
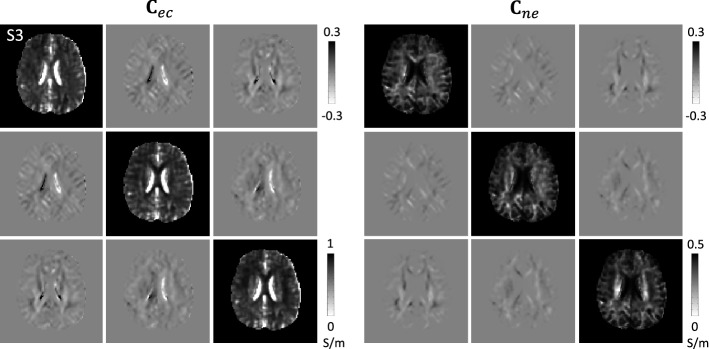
Estimated extracellular conductivity tensor, ${\mathbf{C}}_{ec}$, and intra-neurite conductivity tensor, ${\mathbf{C}}_{ne}$, images from the third imaging slice of the first subject

Figure 6 shows the 3$\times$3 extracellular conductivity tensor, ${\mathbf{C}}_{ec}$, and intra-neurite conductivity tensor, ${\mathbf{C}}_{ne}$, images. To estimate conductivity tensors, we used the water molecule diffusion tensors with the *b* value of 1000 s/$\hbox {mm}^2$. We fixed the principal diffusion direction (eigenvector corresponding to the maximum eigenvalue of the diffusion tensor) and solved the equations (16) and (17) to obtain the extracellular diffusion tensor, ${\mathbf{D}}_{ec}$, and intra- neurite diffusion tensor, ${\mathbf{D}}_{ne}$, images. Using these diffusion tensors, we reconstructed the extracellular conductivity tensor, ${\mathbf{C}}_{ec}$, and intra-neurite conductivity tensor, ${\mathbf{C}}_{ne}$, in (18) and (19), respectively.

Table 3 summarizes the estimated values of high frequency conductivity, extracellular ion concentration, extracellular conductivity, and intra-neurite conductivity of each subject. In Table 3, longitudinal (L), transverse (T), and average (A) white matter conductivity values were found by computing the principal eigenvalue, the mean of the other two eigenvalues, and the mean of all eigenvalues of the conductivity tensor over all region, respectively.

**Table 3:** Estimated values of high-frequency conductivity, $\sigma _H$, extracellular ion concentration, ${\bar{c}}_{ec}$, extracellular conductivity, and intra-neurite conductivity within CSF, white matter, and gray matter regions

	$\sigma _H$	${\bar{c}}_{ec}$	Extracellular conductivity			Intra-neurite conductivity		
Subject 1								
CSF	1.43 ± 0.70	0.57 ± 0.25		1.35 ± 0.72			0.02 ± 0.02	
GM	0.51 ± 0.30	0.45 ± 0.24		0.29 ± 0.20			0.07 ± 0.05	
WM	0.40 ± 0.19	0.39 ± 0.16	L	T	A	L	T	A
			0.28 ± 0.22	0.15 ± 0.11	0.19 ± 0.14	0.19 ± 0.10	0.10 ± 0.04	0.13 ± 0.06
Subject 2								
CSF	1.58 ± 0.67	0.63 ± 0.29		1.49 ± 0.66			0.02 ± 0.03	
GM	0.53 ± 0.26	0.46 ± 0.22		0.30 ± 0.19			0.08 ± 0.05	
WM	0.39 ± 0.15	0.40 ± 0.15	L	T	A	L	T	A
			0.25 ± 0.15	0.13 ± 0.08	0.17 ± 0.10	0.22 ± 0.10	0.10 ± 0.04	0.14 ± 0.06
Subject 3								
CSF	1.51 ± 0.86	0.60 ± 0.34		1.41 ± 0.85			0.03 ± 0.03	
GM	0.55 ± 0.32	0.49 ± 0.28		0.31 ± 0.20			0.07 ± 0.05	
WM	0.39 ± 0.17	0.41 ± 0.16	L	T	A	L	T	A
			0.26 ± 0.17	0.13 ± 0.07	0.17 ± 0.10	0.21 ± 0.12	0.11 ± 0.05	0.14 ± 0.07

The high-frequency conductivity values in CSF regions were between 1.43 and 1.58 S/m and the extracellular conductivity values were between 1.35 and 1.49 S/m. Note SANDI model does not take into account CSF compartment. For this reason, a slight difference between high-frequency conductivity values and extracellular conductivity values was found in CSF regions.

For GM regions, the high-frequency conductivity values were between 0.51 and 0.55 S/m and the extracellular conductivity values were in the range of 0.29 to 0.31 S/m. The extracellular conductivity values were higher than the neurite conductivity values (0.07–0.08 S/m in GM), as expected.

We found longitudinal WM extracellular conductivity values of 0.25–0.28 S/m. Transverse WM extracellular conductivity values were between 0.13 and 0.15 S/m. The average ratio between longitudinal conductivity values and transverse conductivity values was 1.87–2.00. For WM regions, longitudinal intra-neurite conductivity values were 0.19 –0.22 S/m and transverse intra-neurite conductivity values were 0.10–0.11 S/m.

The extracellular conductivity values in GM regions were higher than the average extracellular conductivity values in WM regions, whereas the average intra-neurite conductivity values in WM regions were always higher than the intra-neurite conductivity values in GM regions.

**Fig. 7:**
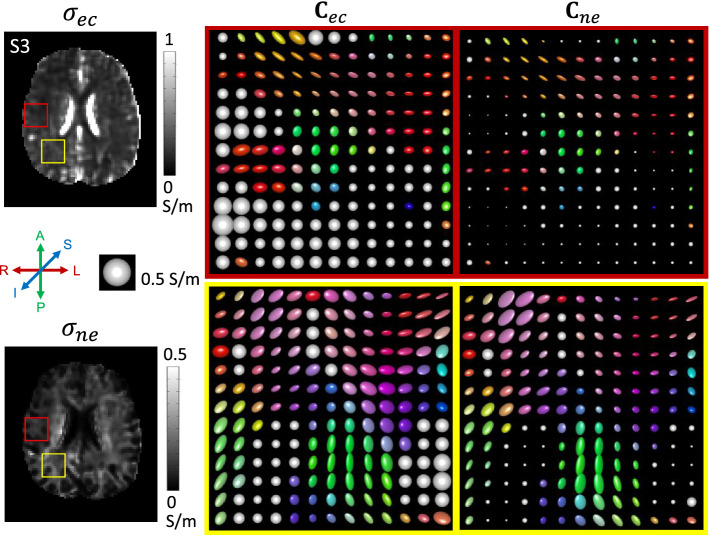
Two chosen rectangular ROIs are shown in extracellular conductivity, $\sigma _{ec}$, and intra-neurite conductivity, $\sigma _{ne}$, images. Estimated extracellular conductivity tensor, ${\mathbf{C}}_{ec}$, and intra-neurite conductivity tensor, ${\mathbf{C}}_{ne}$, images represented by tri-axial ellipsoids, respectively. The radii of each ellipsoid are proportional to the eigenvalues and their axes are oriented along the directions of eigenvectors. For comparison, an isotropic tensor image with conductivity of 0.5 S/m is located in the center left. The colors of ellipsoids indicate the orientation of principle eigenvector

Figure 7 shows the estimated extracellular conductivity tensor, ${\mathbf{C}}_{ec}$, and intra-neurite conductivity tensor, ${\mathbf{C}}_{ne}$, images represented by tri-axial ellipsoids, respectively, in the rectangular ROIs shown in extracellular conductivity, $\sigma _{ec}$, and intra-neurite conductivity, $\sigma _{ne}$, images. Since all of the conductivity tensors shared the same eigenvectors from the diffusion tensor, their orientations were same. The radii of each ellipsoid are proportional to the eigenvalues and their axes are oriented along the directions of eigenvectors. As expected, the volume of intra-neurite conductivity ellipsoids appeared larger in WM regions.

## Discussion

Literature conductivity values including those obtained from direct impedance measurements (IM), MREPT and diffusion tensor MR electrical impedance tomography (DT-MREIT), are summarized in Table 4. The conductivity values of brain tissues heavily depend on the biological tissue structures, participant’s age and pathology, frequency, and the measurement conditions (in vivo, ex vivo, and in vitro) [26–28]. Without external injection currents and using conventional MR pulse sequences minimizing magnetic field inhomogeneity, MREPT is a promising research area for practical clinical medical devices. Comparing to MREPT, by injecting a dc current into the imaging subject, MREIT can reconstruct images of the internal low-frequency conductivity distribution. In this paper, we proposed a new way to decompose electrical properties in each compartment (extracellular, intra-neurite, and soma compartments) from the reconstructed high-frequency conductivity using MREPT technique and the micro-structural parameters using SANDI model.

**Table 4:** Literature conductivity values

Literature conductivity valuesCSF	GM	WM			Method	
		L	T	A		
–	0.69–0.82	–	–	0.30–0.63	MREPT	Human, 64–300 MHz [26]
–	0.70	–	–	0.39	IM	Ovine, 131 MHz [29]
1.71 ± 0.30	0.47 ± 0.24	–	–	0.22 ± 0.14	Meta-analysis	Human, <1 kHz [30]
1.79	-	–	–	–	IM	Human, 10 Hz [28]
1.58, 1.53	0.29, 0.24	0.39, 0.49	0.13, 0.17	0.22, 0.28	DT-MREIT	Human, 10 Hz [31]
1.67	-	0.47	0.08	-	IM	Cat(spinal cord), 5–10 Hz [32]

From the intrinsic noise in the MR measurements, MREPT reconstruction techniques have been proposed to improve the quality of the high-frequency conductivity map [33–36]. The high conductivity values in Table 3 using the reconstruction algorithm in [1] also depend on the first term in (3), which is the diffusion term to stabilize the solution. The diffusion term acts as a low-pass filter, leading to some blurring of the final high-conductivity maps.

Because SANDI model does not take into account CSF compartment, a slight difference between the high-frequency conductivity values and the extracellular conductivity values was found in CSF region as was described in Result section. SANDI model estimates the orientation-independent features of microstructure using the direction-averaged DWI signals. In future studies, it is worth exploring a new model that take into account CSF compartment. In the SANDI model, the direction averaged signals using many uniformly distributed directions are advantageous to estimate the microstructural parameters since the DWI data is noisy at a high-*b* value. Although the accuracy of estimated parameters can be improved as the diffusion directions are increase, the numbers of diffusion directions were 30 and 15 for the phantom and human experiment, respectively, to recover the low-frequency electrical properties within a feasible MR scanning time.

At the low frequency, the internal electrical current flow caused by external current stimulation occurs only in the extracellular space and CSF, excluding the intracellular space, due to the insulation properties of cell membrane [27, 37, 38]. Despite extensive researches for the electrical properties in ECS, the reported low frequency conductivity values do not match and show considerable standard deviation. Recently, a meta-analysis of reported human head electrical conductivity values at low frequency (< 1 kHz) provides a recommended value estimated under suitable and realistic conditions, in which data acquisition techniques were categorized into five groups including directly applied current and MREIT. In Table 4, the reference values for low-frequency conductivity were 1.71 ± 0.3 (CSF), 0.47 ± 0.24 (GM), and 0.22 ± 0.14 S/m (WM) with broadly similar results to ours [30].

At fairly low frequencies, the conductivity of brain tissue, particularly white matter, is known to be anisotropic [32]. Comparing to the anisotropicity of intra-neurite compartment, the average ratio between longitudinal WM extracellular conductivity values and transverse WM conductivity values of 1.87–2.00 found here were lower than 3 and 5.9 reported in [31, 32], respectively. The extracellular conductivity values in GM and WM regions exhibited much stronger frequency dependencies compared to CSF region, because of their complicated tissue structures. More rigorous analysis including geometric and viscous components of the tortuosity of ECS will be needed in the future work.

In this paper, the acquired DWIs are combined because SANDI model assume an isotropic diffusion to distinguish the intra- and extra-cellular diffusion signals. We focus on separating the microscopic parameters for extracellular space from the multiple DWI data. The recovering procedure for the low-frequency electrical properties is very complex and requires further developments, including measurement techniques, noise artifact reduction, main magnetic field inhomogeneity artifact correction, and more reliable models to separate the extracellular and intracellular compartments.

To compensate the difficulties of measuring DWI data for higher *b*-values, we adopt the hypothesis that the DWI data corresponding to the *b*-value range (1000-3600 s/$\hbox {mm}^2$) reflects distinguishable diffusion signals between the soma and the extracellular space. To avoid the ill-posedness to determine the six unknowns, $f_{in}, f_{ec}, D_{in}, D_{is}, D_{ec},$ and $r_s$, in the two compartment model (5) from the smoothly decayed exponential curves, the parameter of $D_{is}$ was fixed as 2 $\times 10^{-3}$ $\hbox {mm}^2$/sec. However, the determination of parameters by matching the observed DWI data for M*b*-DWI data and SANDI model was still very sensitive to measured noise artifacts.

As described in the above, there are several problems to overcome, especially including the follows

- SANDI model is not yet suitable for clinical MRI scanner because it requires high *b*-values (higher than 3000 s/$\hbox {mm}^2$) DWI and relatively many diffusion directions to apply direction averaged DWI signals.
- To estimate the extracellular ion concentration, ${{\bar{c}}}_{ec}$, it is assumed that the intra-neurite and soma ion concentrations are the same. Moreover, the ratio of ion concentrations in the intra- and extra-compartments is assumed as a fixed constant, $\beta =0.41$ for human experiments. Since the ratio $\beta$ depends on the apparent ion concentration, $\beta$ cannot be fixed as a constant in the whole brain region. However, no method to experimentally estimate of the human brain is available.

Nevertheless, the method of extracting low-frequency electrical properties in a non-invasive way from the high-frequency conductivity is critical for clinical usefulness.

Electrical brain stimulation (EBS) techniques, such as transcranial direct current stimulation (tDCS) and deep brain stimulation (DBS), are promising treatments for human disorders [39–43]. Since there is no clear explanation for the mechanism, EBS studies have relied on computational modeling using reference conductivity values in the whole brain region. The proposed electrical property decomposition from the high-frequency conductivity can be a promising work for the EBS techniques.

## Conclusion

We have proposed a method to decompose the electrical properties in the extracellular, intra-neurite, and soma compartments from the high-frequency conductivity map, reconstructed by solving the electro-magnetic equation with measured B1 phase signals. By decomposing the electrical conductivity into the product of mobility and charged carrier concentrations, voxel-wise micro-structures including the extracellular volume fraction and diffusivity were investigated using SANDI model by analyzing the M*b*-DWI data based on a two compartment model. In the SANDI model, to distinguish the intra-soma compartment from the mixed diffusivity signals in ECS, the Gaussian phase distribution approximation of the tail was used. To determine the micro-structural parameters in each separated compartment using M*b*-DWI data, a machine learning algorithm, random forests, was used by constructing a multitude of decision trees. Combining with the predicted DWI data and SANDI model, we separated the extracellular conductivity from the high-frequency conductivity using the decomposed micro-structural diffusion parameters. To verify the proposed method, we conducted human experiments to verify that the proposed method recovered the low-frequency electrical properties using a routine protocol sequence of MRI scan.

## Methods

### High-frequency conductivity at Larmor frequency using B1 phase map

The electrical conductivity of biological tissue as a function of frequency is complicated by the anisotropic nature of tissue, non-homogeneous natures in the extracellular and intracellular compartments, and randomly distributed cells sizes. The high-frequency conductivity is dominantly isotropic because the electrical current flow tends to pass through the cell membrane.

For the positive (negative) rotating component of the transmit B1 field $B_1^+={\left| \,B_1^+\,\right| }e^{i\varphi ^+}$ ($B_1^-={\left| \,B_1^-\,\right| }e^{i\varphi ^-}$), by assuming $\sigma _H \gg \omega \epsilon _H$, a phase-based MREPT formula has been proposed and conducted for numerous clinical studies [44–47]:

2 $$\begin{aligned} \nabla \varphi ^{tr}\cdot \nabla \tau _H+\tau _H\nabla ^2\varphi ^{tr}-2\omega \mu _0=0 \end{aligned}$$

where $\tau _H$ denotes $\frac{1}{\sigma _H}$ and $\varphi ^{tr}=\varphi ^++\varphi ^-$ is the measurable transceive phase using MRI [1].

Since the reaction–diffusion equation is sensitive to the measured noise, to stabilize the formula (2), after adding an artificial diffusion term, the equation (2) leads to

3 $$\begin{aligned} -c\nabla ^2\tau _H+\nabla \varphi ^{tr}\cdot \nabla \tau _H+\tau _H\nabla ^2\varphi ^{tr}=2\omega \mu _0 \end{aligned}$$

where *c* is a constant diffusion coefficient.

### Detection of the micro-structures of biological tissues based on intracellular and extracellular compartments

For the model-based micro-structure imaging based on the tissue micro architecture in the brain, the model parameters for the intracellular and extracellular compartments are associated with specific tissue micro-structure characteristics from M*b*-DWI data [7–9]. The signal intensity $S_b$ by applying a diffusion encoding gradient is given by

4 $$\begin{aligned} S_b=S_0\exp (-bD) \end{aligned}$$

where $S_0$ is the signal obtained without diffusion gradient and *b* denotes the diffusion-weighting factor. To distinguish the diffusion signals from ECS and the cell bodies of any brain cell type (collectively named soma) [9], SANDI model proposes the following compartment model of brain tissue micro-structure:

5 $$\begin{aligned} S_b=S_0\left( f_{ic}\left( f_{in}A_{in}(b)+f_{is}A_{is}(b)\right) +f_{ec}A_{ec}(b)\right) \end{aligned}$$

where $f_{ic}$ and $f_{ec}$ are the intracellular and extracellular volume fractions, $f_{ic}+f_{ec}=1$; $f_{in}$ and $f_{is}$ are the neurite and soma relative volume fractions in the intracellular compartment, $f_{in}+f_{is}=1$; $A_{in}$ and $A_{is}$ are the normalized signals for restricted diffusion within neurites and soma, respectively, and $A_{ec}$ is the normalized signal of the extracellular compartment. To investigate the complicated micro-structural model (5), some assumptions are applied to each parameter. The diffusion of water molecules associated with the extracellular compartment is modeled as isotropic Gaussian diffusion:

6 $$\begin{aligned} A_{ec}(b)=e^{-bD_{ec}} \end{aligned}$$

The diffusion signals $A_{in}$ from neurites are assumed as a collection of sticks (long thin cylinders). The direction-averaged DWI signal $A_{in}$ is computed as [11]

7 $$\begin{aligned} A_{in}(b)=\sqrt{\frac{\pi }{4bD_{in}}} {erf}\left( \sqrt{bD_{in}}\right) \end{aligned}$$

where *erf* is the error function. The signal contribution, $A_{is}$, for the inra-soma compartment is computed from the Gaussian phase distribution (GPD) approximation of the tail:

8 $$\begin{aligned} A_{is}(b)=\exp \left\{ -\frac{2(\gamma G)^2}{D_{is}}\left( \sum _{m=1}^\infty \frac{\alpha ^{-4}_m}{\alpha ^2_mr^2_s-2}\right) \left( 2\delta -\Psi \right) \right\} \end{aligned}$$

where

$$\begin{aligned} \Psi =\frac{2+e^{-\alpha ^2_mD_{is}(\Delta -\delta )}-2e^{-\alpha ^2_mD_{is}\delta } -2e^{-\alpha ^2_mD_{is}\Delta }+e^{-\alpha ^2_mD_{is}(\Delta +\delta )}}{\alpha ^2_mD_{is}}, \end{aligned}$$

$D_{is}$ is the bulk diffusivity of water in soma, $\alpha _m$ is the *m*-th root of the Bessel equation $(\alpha r_s)^{-1}J_{\frac{3}{2}}(\alpha r_s)=J_{\frac{5}{2}}(\alpha r_s)$.

The total unknown parameters to be determined are $f_{in}, f_{ec}, D_{in}, D_{is}, D_{ec},$ and $r_s$. To avoid the ill-posedness to determine the six unknowns from the smoothly decayed exponential curves, typically the parameter of $D_{is}$ is fixed as 2 $\times 10^{-3}$ $\hbox {mm}^2$/sec [9].

### Model-parameter estimation using random forest regression

Random forest is a popular machine learning algorithm using the bootstrap aggregating (bagging) with a tree model as the base model. Random forest regression is an ensemble supervised learning method that combines bagging decision trees with random subset sampling of the predictors for constructing each node split [22]. To perform the random forest regression, each individual decision tree produces a prediction individually and then predictions of all decision trees are combined to generate a prediction of the ensemble. The number of trees can be adapted to find the desired trade-off between accuracy and computational efficiency of the detection process.

**Fig. 8:**
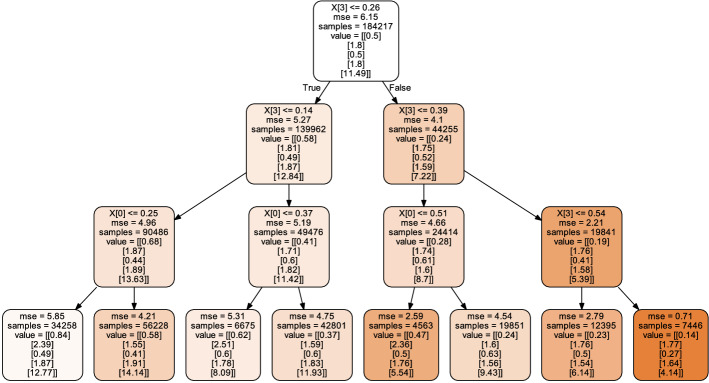
Sample visualization for a small decision tree

The five parameters $f_{in}, f_{ec}, D_{in}, D_{ec},$ and $r_s$ in (5) are estimated by random forest regression using the scikit-learn python toolkit [48] as in [9]. We generated $13^5$ synthetic signals using the model (5) with $13^5$ combinations of the five parameters chosen uniformly distributed within the intervals: $f_{in},\,\,f_{ec} \in [0.01,0.99]$, $D_{in},\,\,D_{ec} \in [0.1, 3]~ \times 10^{-3}$ $\hbox {mm}^2$/sec, and $r_s \in [3, 20]~ \mu$m. We added rician-distributed noise to the synthetic signals. We split the synthetic signals into random train and test subsets with test size of 20%. Hyperparameter selection in random forest regression was performed using a grid search for the number of decision trees (150, 180, 200, 230, 250, 280, 300 and 330 trees). The remaining hyperparameters were left to default in scikit-learn toolkit. By comparing the mean squared errors obtained from the different number of trees, the final random forest regressor was built with 300 trees. One decision tree in the random forest regressor is displayed in Fig. 8. To produce an understandable image, the depth of the decision tree was limited to three.

### High-frequency conductivity decomposition

The recovered high-frequency conductivity $\sigma _H$ at Larmor frequency, obtained by solving the equation (3), can be decomposed as the following compartment mode:

9 $$\begin{aligned} \sigma _H=\left( \sigma _{ne}+\sigma _{so}\right) +\sigma _{ec} \end{aligned}$$

where $\sigma _{ne}$ and $\sigma _{so}$ denote the conductivity in the intra-neurite and soma compartments, respectively, and $\sigma _{ec}$ is the conductivity in the extracellular compartment. At each compartment, the apparent conductivities $\sigma _{ne}$, $\sigma _{so}$, and $\sigma _{ec}$ are expressed as the sum of products of concentration, charge carrier mobility, and the charger of carrier. For simplicity of notation, we write $f_{ne}$ and $f_{so}$ instead of $f_{ic} f_{in}$ and $f_{ic}f_{is}$, respectively:

10 $$\begin{aligned} \sigma _{ne}&=f_{ne}\sum _{j=1}^{N_{n}}z_n^jqc^n_jm^n_j =f_{ne}\sum _{j=1}^{N_{n}}z_n^jqc^n_j\left( \frac{r_wq}{r_jk_BT}\right) D_{in}\nonumber \\&=f_{ne}{\bar{c}}_{in}D_{in} \end{aligned}$$

11 $$\begin{aligned} \sigma _{so}&=f_{so}\sum _{j=1}^{N_{s}}z_s^jqc^s_jm^s_j =f_{so}\sum _{j=1}^{N_{s}}z_s^jqc^s_j\left( \frac{r_wq}{r_jk_BT}\right) D_{is}\nonumber \\&=f_{so}{\bar{c}}_{is}D_{is} \end{aligned}$$

12 $$\begin{aligned} \sigma _{ec}&=f_{ec}\sum _{j=1}^{N_{ec}}z_e^jqc^e_jm^e_j =f_{ec}\sum _{j=1}^{N_{ec}}z_e^jqc^e_j\left( \frac{r_wq}{r_jk_BT}\right) D_{ec} \nonumber \\&=f_{ec}{\bar{c}}_{ec}D_{ec} \end{aligned}$$

where ${\bar{c}}_{ec}:=\sum _{j=1}^{N_{ec}}z_e^jqc^e_j\left( \frac{r_wq}{r_j k_BT}\right)$ denote the apparent ion concentrations with respect to the water molecule diffusivity in the extracellular compartments. The other symbols of the physical quantities are as follows: $r_w$ and $r_j$ are the Stoke’s radius of a water molecule and an ion, respectively, $q=1.6\times 10^{-19}$C is the electric charge carried by a single proton, $k_B$ is the Boltzmann constant, and *T* is the absolute temperature. $c^e_j$, $m^e_j$ $z^j_eq$, and $N_{ec}$ are concentration, the charge carrier mobility, the charge of carrier, and the number of electrical charges in the extracellular space, respectively.

By the same argument, $\sigma _{ne}=f_{ne}{\bar{c}}_{in}D_{in}$ and $\sigma _{so}=f_{so}{\bar{c}}_{is}D_{is}$, where ${\bar{c}}_{in}$ and ${\bar{c}}_{is}$ denote the apparent ion concentrations with respect to the water molecule diffusivity in the intra-neurite, and soma compartments, respectively.

### Extracellular diffusion tensor

Diffusion process is sensitive to intracellular, extracellular, and cell density. For a fixed *b* value, the measured non-singular diffusion tensor $\mathbf {D}_b$ can be diagonalized as

13 $$\begin{aligned} \mathbf {D}_b= {\mathcal {S}}_D {\tilde{\mathbf {D}}}_b {\mathcal {S}}^T_D \quad \text{ with }\quad {\tilde{\mathbf {D}}}_b= \left( \begin{array}{ccc} d^b_1 &{} 0 &{} 0 \\ 0 &{}d^b_2 &{} 0 \\ 0 &{} 0 &{} d^b_3 \end{array} \right) \end{aligned}$$

where the column vectors of ${\mathcal {S}}_D$ are the orthonormal eigenvectors of $\mathbf {D}_b$, the superscript *T* denotes the transpose and $d^b_1\ge d^b_2\ge d^b_3$ are the corresponding eigenvalues.

We separate the apparent diffusion tensor $\mathbf {D}_b$ into the extracellular and intracellular compartments:

14 $$\begin{aligned} \mathbf {D}_b=\mathbf {D}_{ec}+\mathbf {D}_{ne}+\mathbf {D}_{so} \end{aligned}$$

where $\mathbf {D}_{ec}$ and $\mathbf {D}_{ne}$ denote the apparent diffusion tensors in ECS and intra-neurite compartment, respectively, and $\mathbf {D}_{so}$ is the isotropic diffusion. By assuming that the diffusion tensors $\mathbf {D}_{ec}$, $\mathbf {D}_{ne}$, and $\mathbf {D}_b$ share the eigenvectors, the intrinsic diffusion tensors $\mathbf {D}_{es}$ and $\mathbf {D}_{ne}$ can be expressed as

15 $$\begin{aligned} \mathbf {D}_{ec}= {\mathcal {S}}_D {\tilde{\mathbf {D}}}_{ec} {\mathcal {S}}^T_D \quad \text{ and }\quad \mathbf {D}_{ne}= {\mathcal {S}}_D {\tilde{\mathbf {D}}}_{ne} {\mathcal {S}}^T_D \end{aligned}$$

where ${\tilde{\mathbf {D}}}_{ec}$ and ${\tilde{\mathbf {D}}}_{ne}$ are the diagonal matrices consist of the eigenvalues of $\mathbf {D}_{ec}$ and $\mathbf {D}_{ne}$, respectively. To translate the estimated intrinsic diffusivities $D_{ec}$ and $D_{in}$ to apparent diffusion tensors in each compartment, we define scale parameters as

16 $$\begin{aligned} \eta _{ec} = f_{ec}\frac{3 D_{ec}}{tr(\mathbf {D}_b)}, \quad \text{ and }\quad \eta _{ne} = f_{ne}\frac{3 D_{in}}{tr(\mathbf {D}_b)} \end{aligned}$$

where $tr(\mathbf {D}_b)=d^b_1+d^b_2+d^b_3$ denotes the trace of $\mathbf {D}_b$. Under the hypothesis that the extracellular diffusion tensor $\mathbf {D}_{ec}$, the diffusion tensor $\mathbf {D}_{ne}$, and the diffusion tensor $\mathbf {D}_b$ share the eigenvectors, we can determine the decomposed diffusion tensors:

17 $$\begin{aligned} \mathbf {D}_{ec}=\eta _{ec} \mathbf {D}_b \quad \text{ and }\quad \mathbf {D}_{ne}=\eta _{ne} \mathbf {D}_b \end{aligned}$$

From the relation (17), the conductivity tensors in ECS and the neurite compartment can be expressed as the following

18 $$\begin{aligned} {\mathbf{C}}_{ec}= {\bar{c}}_{ec} \mathbf {D}_{ec}= {\bar{c}}_{ec} \eta _{ec} \mathbf {D}_{b} \end{aligned}$$

and

19 $$\begin{aligned} {\mathbf{C}}_{ne}= {\bar{c}}_{in} \mathbf {D}_{ne}= {\bar{c}}_{in} \eta _{ne} \mathbf {D}_{b} \end{aligned}$$

### Data processing

The motion, eddy current distortion, and EPI distortion of DWI images were corrected using the DIFFPREP tool of TORTOISE [49, 50]. To reduce the noise artifacts, we used odd echoes of six measured complex MREPT signals to avoid the background phase signal due to the consecutive 180$^\circ$ RF pulses. Since the accumulated noise artifacts in the phase signal is inversely proportional to MR magnitude intensity, $\tilde{{\mathcal {S}}}_k,~k=1,3,5$, the measured phase signal was optimized as a weighted averaging using the weight of [51]

20 $$\begin{aligned} w_k = \frac{|\tilde{{\mathcal {S}}}_k|^2}{|\tilde{{\mathcal {S}}}_1|^2 +|\tilde{{\mathcal {S}}}_2|^2 +|\tilde{{\mathcal {S}}}_3|^2}, ~k=1,3,5 \end{aligned}$$

The procedure of combining the high frequency conductivity and diffusion parameter (e.g. extracellular volume fraction and extracellular diffusivity) requires an accurate registration. Using the co-registration tool of Statistical Parametric Mapping (SPM 12), the diffusion images and the diffusion parameter maps were transformed to the first echo magnitude of MREPT image.

## Data Availability

All data generated or analyzed during this study are included in this published article.

## Abbreviations

M*b*-DWI: multi-*b* diffusion-weighted imaging
MREPT: Magnetic resonance electrical properties tomography
EVF: Extracellular volume fraction
SANDI: Soma and neurite density imaging
ECS: Extracellular space
SS-SE-EPI: Single-shot spin-echo echo planner imaging
CSF: Cerebrospinal fluid
GM: Gray matter
WM: White matter
IM: Impedance measurements
DT-MREIT: Diffusion tensor magnetic resonance electrical impedance tomography
EBS: Electrical brain stimulation
tDCS: Transcranial direct current stimulation
DBS: Deep brain stimulation

## References

[1] Gurler N, Ider YZ (2017). Gradient-based electrical conductivity imaging using MR phase. Magn Reson Med.

[2] Katscher U, Voigt T, Findeklee C, Vernickel P, Nehrke K, Doessel O (2009). Determination of electric conductivity and local SAR via B1 mapping. IEEE Trans Med Imaging.

[3] Voigt T, Katscher U, Doessel O (2011). Quantitative conductivity and permittivity imaging of the human brain using electric properties tomography. Magn Reson Med.

[4] Lee J, Shin J, Kim DH (2016). MR-based conductivity imaging using multiple receiver coils. Magn Reson Med.

[5] Tuck DS, Wedeen VJ, Dale AM, George JS, Belliveau JW (2001). Conductivity tensor mapping og the human brain using diffusion tensor MRI. Proc Natl Acad Sci.

[6] Sajib SZ, Kwon OI, Kim HJ, Woo EJ (2018). Electrodeless conductivity tensor imaging (cti) using mri: basic theory and animal experiments. Biomed Eng Lett.

[7] Alexander DC, Dyrby TB, Nilsson M, Zhang H (2019). Imaging brain microstructure with diffusion mri: practicality and applications. NMR Biomed.

[8] Novikov DS, Veraart J, Jelescu IO, Fieremans E (2018). Rotationally-invariant mapping of scalar and orientational metrics of neuronal microstructure with diffusion mri. NeuroImage.

[9] Palombo M, Ianus A, Guerreri M, Nunes D, Alexander DC, Shemesh N, Zhang H. Sandi: a compartment-based model for non-invasive apparent soma and neurite imaging by diffusion mri. NeuroImage. 2020;116835. 10.1016/j.neuroimage.2020.116835 PMC8543044 32289460

[10] Panagiotaki E, Schneider T, Siow B, Hall MG, Lythgoe MF, Alexander DC (2012). Compartment models of the diffusion mr signal in brain white matter: a taxonomy and comparison. Neuroimage.

[11] Kaden E, Kruggel F, Alexander DC (2016). Quantitative mapping of the per-axon diffusion coefficients in brain white matter. Magn Reson Med.

[12] Le Bihan D, Urayama S, Aso T, Hanakawa T, Fukuyama H (2006). Direct and fast detection of neuronal activation in the human brain with diffusion MRI. Proc Natl Acad Sci.

[13] Le Bihan D (2008). Intravoxel incoherent motion perfusion MR imaging: a wake-up call. Radiology.

[14] Bennett KM, Schmainda KM, Bennett R, Rowe DB, Lu H, Hyde JS (2003). Characterization of continuously distributed cortical water diffusion rates with a stretched-exponential model. Magn Reson Med.

[15] Jensen JH, Helpern JA, Ramani A, Lu H, Kaczynski K (2005). Diffusional kurtosis imaging: the quantification of non-gaussian water diffusion by means of magnetic resonance imaging. Magn Reson Med.

[16] Zhou XJ, Gao Q, Abdullah O, Magin RL (2010). Studies of anomalous diffusion in the human brain using fractional order calculus. Magn Reson Med.

[17] Lee MB, Jahng G-H, Kim HJ, Woo EJ, Kwon OI (2020). Extracellular electrical conductivity property imaging by decomposition of high-frequency conductivity at larmor-frequency using multi-b-value diffusion-weighted imaging. PLoS ONE.

[18] Katoch N, Choi BK, Sajib SZ, Lee EA, Kim HJ, Kwon OI, Woo EJ. Conductivity tensor imaging of in vivo human brain and experimental validation using giant vesicle suspension. IEEE Transactions on Medical Imaging. 2018. 10.1109/TMI.2018.2884440 30507528

[19] Jahng G-H, Lee MB, Kim HJ, Woo EJ, Kwon O-I (2021). Low-frequency dominant electrical conductivity imaging of in vivo human brain using high-frequency conductivity at larmor-frequency and spherical mean diffusivity without external injection current. NeuroImage.

[20] Ho TK. Proceedings of the 3rd international conference on document analysis and recognition. Random Decision Forests; 1995, p. 278–282.

[21] Ho TK (2002). A data complexity analysis of comparative advantages of decision forest constructors. Pattern Anal Appl.

[22] Breiman L (2001). Random forests. Mach Learn.

[23] Moscho A, Orwar O, Chiu DT, Modi BP, Zare RN (1996). Rapid preparation of giant unilamellar vesicles. Proc Natl Acad Sci.

[24] Lin W, Wehrli FW, Song HK (2005). Correcting bulk in-plane motion artifacts in mri using the point spread function. IEEE Trans Med Imaging.

[25] Penny WD, Friston KJ, Ashburner JT, Kiebel SJ, Nichols TE (2011). Statistical parametric mapping: the analysis of functional brain images.

[26] Hancu I, Liu J, Hua Y, Lee S-K (2019). Electrical properties tomography: available contrast and reconstruction capabilities. Magn Reson Med.

[27] Gabriel C, Peyman A, Grant E (2009). Electrical conductivity of tissue at frequencies below 1 mhz. Phys Med Biol.

[28] Baumann SB, Wozny DR, Kelly SK, Meno FM (1997). The electrical conductivity of human cerebrospinal fluid at body temperature. IEEE Trans Biomed Eng.

[29] Gabriel C, Gabriel S. Compilation of the dielectric properties of body tissues at RF and microwave frequencies; 1997. http://niremf.ifac.cnr.it/docs/DIELECTRIC/home.html. Accessed 3 Aug 2020.

[30] McCann H, Pisano G, Beltrachini L (2019). Variation in reported human head tissue electrical conductivity values. Brain Topogr.

[31] Chauhan M, Indahlastari A, Kasinadhuni AK, Schär M, Mareci TH, Sadleir RJ (2017). Low-frequency conductivity tensor imaging of the human head in vivo using DT-MREIT: first study. IEEE Trans Med Imaging.

[32] Ranck JB, BeMent SL (1965). The specific impedance of the dorsal columns of cat: an anisotropic medium. Exp Neurol.

[33] Sodickson DK, Alon L, Deniz CM, Ben-Eliezer N, Cloos M. Sodickson LA, Collins CM, Wiggins GC, Novikov DS. Generalized local maxwell tomography for mapping of electrical property gradients and tensors. In: Proceedings of 21th annual meeting ISMRM, Salt Lake City, USA, 2013;vol. 4175.

[34] Hafalir FS, Oran OF, Gurler N, Ider YZ (2014). Convection-reaction equation based magnetic resonance electrical properties tomography (cr-mrept). IEEE Trans Med Imaging.

[35] Marques JP, Sodickson DK, Ipek O, Collins CM, Gruetter R (2015). Single acquisition electrical property mapping based on relative coil sensitivities: a proof-of-concept demonstration. Magn Reson Med.

[36] Ropella KM, Noll DC (2017). A regularized, model-based approach to phase-based conductivity mapping using mri. Magn Reson Med.

[37] Gabriel S, Lau R, Gabriel C (1996). The dielectric properties of biological tissues: Ii. measurements in the frequency range 10 hz to 20 ghz. Phys Med Biol.

[38] Miklavčič D, Pavšelj N, Hart FX. Electric properties of tissues. Wiley encyclopedia of biomedical engineering 2006.

[39] Brunoni AR, Moffa AH, Fregni F, Palm U, Padberg F, Blumberger DM, Daskalakis ZJ, Bennabi D, Haffen E, Alonzo A, Loo CK (2016). Transcranial direct current stimulation for acute major depressive episodes: meta-analysis of individual patient data. Br J Psychiatry.

[40] Eichelbaum S, Dannhauer M, Hlawitschka M, Brooks D, Knösche TR, Scheuermann G (2014). Visualizing simulated electrical fields from electroencephalography and transcranial electric brain stimulation: a comparative evaluation. NeuroImage.

[41] Rosa MA, Lisanby SH (2012). Somatic treatments for mood disorders. Neuropsychopharmacology.

[42] Beloucif S (2013). Informed consent for special procedures: electroconvulsive therapy and psychosurgery. Curr Opin Anaesthesiol.

[43] Benabid AL (2015). Neuroscience: spotlight on deep-brain stimulation. Nature.

[44] Balidemaj E, van Lier AL, Crezee H, Nederveen AJ, Stalpers LJ, Van Den Berg CA (2015). Feasibility of electric property tomography of pelvic tumors at 3t. Magn Reson Med.

[45] Kim S-Y, Shin J, Kim D-H, Kim MJ, Kim E-K, Moon HJ, Yoon JH (2016). Correlation between conductivity and prognostic factors in invasive breast cancer using magnetic resonance electric properties tomography (mrept). Eur Radiol.

[46] Tha KK, Katscher U, Yamaguchi S, Stehning C, Terasaka S, Fujima N, Kudo K, Kazumata K, Yamamoto T, Van Cauteren M (2018). Noninvasive electrical conductivity measurement by mri: a test of its validity and the electrical conductivity characteristics of glioma. Eur Radiol.

[47] Mori N, Tsuchiya K, Sheth D, Mugikura S, Takase K, Katscher U, Abe H (2019). Diagnostic value of electric properties tomography (ept) for differentiating benign from malignant breast lesions: comparison with standard dynamic contrast-enhanced mri. Eur Radiol.

[48] Pedregosa F, Varoquaux G, Gramfort A, Michel V, Thirion B, Grisel O, Blondel M, Prettenhofer P, Weiss R, Dubourg V (2011). Scikit-learn: machine learning in python. J Mach Learn Res.

[49] Pierpaoli C, Walker L, Irfanoglu M, Barnett A, Basser P, Chang L, Koay C, Pajevic S, Rohde G, Sarlls J. et al. Tortoise: an integrated software package for processing of diffusion mri data. In: ISMRM 18th Annual Meeting, 2010;vol. 1597.

[50] Irfanoglu MO, Nayak A, Jenkins J, Pierpaoli C. Tortoise v3: Improvements and new features of the nih diffusion mri processing pipeline. In: Proceedings of the 25th annual meeting of ISMRM presented at the international society for magnetic resonance in medicine; 2017.

[51] Kwon OI, Jeong WC, Sajib SZ, Kim HJ, Woo EJ, Oh TI (2014). Reconstruction of dual-frequency conductivity by optimization of phase map in mreit and mrept. Biomed Eng Online.

